# Factors associated with cerebral palsy among children in Hawassa University comprehensive specialized hospital: Case-control study

**DOI:** 10.1371/journal.pone.0333406

**Published:** 2025-09-26

**Authors:** Bethelhem Bashe, Desalegn Dawit Assele, Worku Ketema, Mulugeta Sitot Shibeshi

**Affiliations:** 1 Department of Pediatrics and Child Health, College of Medicine and Health Sciences, Hawassa University, Hawassa, Ethiopia; 2 Department of Public Health, College of Medicine and Health Sciences, Hawassa University, Hawassa, Ethiopia; 3 Pediatric Neurology Division, Department of Pediatrics and Child Health, College of Medicine and Health Sciences, Hawassa University, Hawassa, Ethiopia; Arba Minch University, ETHIOPIA

## Abstract

**Background:**

Cerebral palsy is a frequent physical disability of childhood, causing motor impairment, sensory impairment, cognitive and behavioral issues, and secondary musculoskeletal deformities, with a global incidence of 1–4 per 1,000 children. It significantly impacts children’s quality of life and imposes an economic burden on families and healthcare systems. There is limited evidence of the risk factors of cerebral palsy in Ethiopia, including in the study setting. We investigated factors associated with cerebral palsy among children attending Hawassa University Comprehensive Specialized Hospital.

**Methods:**

An institution-based, unmatched case-control study was conducted among children who visited Hawassa University Comprehensive Specialized Hospital from January 2019 to December 2023. Consecutive cases were recruited until the required sample size was reached, and controls were randomly selected. Data were extracted from 80 cases and 160 control charts. Binary logistic regression analysis was used to identify risk factors for cerebral palsy. An adjusted odds ratio with a 95% confidence interval was reported to show the strength of the association. The significance of the association was declared at a p-value < 0.05. The goodness-of-fit model was checked by the Hosmer and Lemeshow test.

**Results:**

A total of 240 participants (80 cases and 160 controls) were enrolled in the study. Maternal infection during pregnancy [AOR:4.1; 95%; 1.39, 12.1], low birth weight [AOR:4.1; 95%; 1.49, 11.2], prolonged labor [AOR:3.2; 95%;1.47, 7.00], history of perinatal asphyxia [AOR: 2.65; 95%;1.06, 6.65], and central nervous system infection during infancy [AOR:3.4; 95%; 1.21, 9.64] were risk factors for cerebral palsy.

**Conclusion:**

Perinatal asphyxia, maternal infection, low birth weight, prolonged labor, and CNS infection during infancy are significantly associated with cerebral palsy. Public health education should promote awareness about cerebral palsy, encourage antenatal care, and educate healthcare professionals on emergency obstetrics and newborn care. Appropriate measures should be taken to reduce the incidence of CNS infections during infancy.

## Introduction

Cerebral palsy (CP) is a movement and posture disorder caused by a non-progressive lesion to an immature brain, occurring in utero, near delivery, or within the first three years of life [1]. CP is the primary cause of childhood physical disability, causing motor impairment, sensory disturbances, cognitive and behavioral issues, and secondary musculoskeletal problems [2]. It is characterized by motor deficits, causing ambulation delays and self-care difficulties, and can cause intellectual disability, seizure disorder, and visual, hearing, and pulmonary problems [3]. CP is classified into spastic, ataxic, dyskinetic, hypotonic, and mixed forms, with quadriplegic CP being the most severe type [4]. The global prevalence of CP is 1–4 per 1,000 children, with a male-to-female ratio of 1.4:1 [5]. The prevalence has marginally risen due to the survival of very premature babies, who develop CP at 15 per 100 [6].

The causes of CP are often multifactorial and heterogeneous, with identifiable causes often not being identified in many cases [6,7]. Studies indicate that prenatal risk factors are more prevalent in developed countries, while postnatal causes are more prevalent in developing countries [8,9]. Perinatal risk factors include prematurity, intracranial bleeding, perinatal asphyxia, kernicterus, low birth weight, meningoencephalitis, hypoglycemia, meconium aspiration syndrome, and neonatal seizures [10–14]. Further, advanced maternal age is linked to CP, as older women are more susceptible to conditions like obesity, gestational diabetes, and hypertension, which increase the risk of preterm birth, low birth weight, fetal distress, and abnormal perinatal outcomes [2].

CP is linked with various developmental disabilities like epilepsy, intellectual, visual, hearing, speech, and behavioral abnormalities [6]. Hearing impairments and swallowing disorders are some of the comorbidities that can significantly impact children’s quality of life. Early treatment is necessary to prevent profound communication impairments, and management of the etiology of CP can improve patient care and quality of life in general [15]. However, most children with cerebral palsy in Africa face social, economic, and psychological challenges due to limited access to healthcare facilities, specialists, and adaptive equipment. High social stigma exacerbates this treatment gap, leading families to avoid seeking treatment and causing devastating impacts on victims and their families [15].

CP cannot be cured, and therefore interdisciplinary treatment interventions are used to manage it, including physical, developmental, medical, surgical, and technical interventions. Traditional physiotherapy and occupational therapy are widely used, while medical interventions like botulinum toxin injections and phenol injections are beneficial. Orthopedic surgery and thermoplastic materials are used in children [16]. Hippo therapy is a treatment involving activities with horses and equines to enhance gross motor function in individuals with cerebral palsy, improving their physical, occupational, and emotional well-being [17].

CP has an enormous financial burden on families, the health care system, and the general economy, encompassing both direct expenses such as treatment and medical care as well as indirect costs like caregivers’ missed wages [18]. Several factors contribute to the extraordinarily high economic burden of CP, including productivity loss, reduced life expectancy, and dependency [19]. Therefore, understanding the etiology and risk factors of CP can lead to the implementation of more preventive measures, benefiting individuals and the community by reducing the number of cases [20]. There is limited evidence about the risk factors of cerebral palsy in Ethiopia, including in the study setting. We investigated the risk factors of cerebral palsy among children attending Hawassa University Comprehensive Specialized Hospital.

## Methods

### Study area

This study was conducted at Hawassa University Comprehensive Specialized Hospital from January 1^st^ to 30^th,^ 2024. HUCSH is one of the teaching hospitals found in Hawassa city, the capital city of the Sidama region, Hawassa city, Ethiopia. Pediatrics and Child Health is one of the departments where around 15,000 pediatric patients are treated per year. The pediatric neurology follow-up clinic operates once weekly. The clinic is attended by a pediatric neurologist, 2–3 pediatric residents, and a nurse to evaluate patients on regular follow-up and is visited by approximately 20–25 children and adolescents aged from 0 to 15 years per week.

### Study design and population

A facility-based unmatched case-control study was conducted. All children aged 2–15 years attending HUCSH between January 2019 and December 2023 were the source population. The study populations for the cases were children aged 2–14 years with a confirmed diagnosis of cerebral palsy, defined as a non-progressive disorder of movement and posture caused by an injury or disturbance in the developing brain, with onset during the prenatal, perinatal, or early postnatal period. The diagnosis was made by a pediatric neurologist. The study populations for controls were children aged 2–14 years who did not have cerebral palsy and had no history or clinical evidence of any chronic neurological or motor disability. For both cases and controls, children were excluded if they were younger than two years of age, had progressive neurological or metabolic disorders, primary neuromuscular disorder, or experienced major acquired brain injury after the neonatal period, such as head trauma or stroke. Those with other chronic neurological or motor conditions, including muscular dystrophy, spina bifida, or severe neurodevelopmental disorders, were also excluded. In addition, children were not considered if their medical charts lacked sufficient information to confirm eligibility or to extract the required exposure and outcome data.

### Sample size and sampling technique

We used Epi Info version 7 statistical software to determine the sample size for an unmatched case-control study, with assumptions including a 95% confidence level, 80% power, a 1:2 case-to-control ratio, and a percentage of exposure among controls and cases (proportion of vacuum delivery section among children). The percent of cases exposed to vacuum delivery (12%) and the percent of controls exposed to vacuum delivery (2%) were taken from a study conducted in Ayder Comprehensive Specialized Referral Hospital [21]. Based on the above assumptions, the sample size was 249 (83 cases and 166 controls). All cases were included in the study, and a systematic sampling method was used to select two controls for each case, with the interval (K^th^) calculated by dividing the average number of controls who visited the hospital in the previous month.

### Study variables

The outcome variable of the study was cerebral palsy. The independent variables were **s**ocio-demographic variables (age, residence), prenatal factors (maternal age, maternal health conditions, prenatal care utilization), intrapartum factors (mode of delivery, duration of labor, perinatal factors (prematurity, low birth weight, birth complications), and postnatal factors (early childhood infections).

### Operational definition

Cerebral palsy (CP) is a brain disorder that appears in infancy or early childhood and permanently affects body movement and muscle coordination [1].

**Perinatal asphyxia** is, according to WHO, failure to initiate breathing/failure to start crying after birth [22].

**Neonatal hyperbilirubinemia** is an increased serum bilirubin level during the neonatal age, which manifests as yellowish discoloration of the body and needs treatment [23].

**Maternal infection**: Clinically or laboratory-confirmed infection during pregnancy, including UTI, chorioamnionitis, sepsis, or other systemic infections [24].

**Prolonged labor**: Labor lasting more than 12 hours for primiparous mothers or more than 8 hours for multiparous women from the onset of active labor to delivery [25].

**CNS infection**: Any documented infection of the central nervous system, including meningitis, encephalitis, or brain abscess, confirmed by clinical assessment, cerebrospinal fluid analysis, or neuroimaging [26].

### Data collection tool and procedure

An English version checklist was developed after reviewing different kinds of literature [2,11,21]. Cerebral palsy was diagnosed by a pediatric neurologist based on clinical assessment, developmental assessment, and neurological examination focusing on motor tone, posture, reflexes, and functional motor skills. Initial identification relied on routine pediatric neurology evaluation, which included a detailed history of pregnancy, delivery, and early developmental milestones, combined with a structured neurological examination. Key clinical tools used were the assessment of motor tone (spasticity, dystonia, or hypotonia), posture, reflexes, gait, and functional motor skills. Children diagnosed or suspected of having CP at the initial evaluation were scheduled for at least one follow-up visit to confirm the stability of motor impairment and to rule out progressive or alternative neurological conditions. Standardized developmental checklists were also used. Data were collected by reviewing patients’ medical charts. The checklist consists of demographic, obstetric-related characteristics, prenatal risk factors, and post-natal risk factors-related variables. Data were collected by two trained medical interns and supervised by one pediatric resident.

### Data quality assurance

A pre-test was done on 5% (4 cases and 8 controls) of the study sample of children. Before the data collection period, one day of training was given to data collectors and supervisors. On-site supervision was done to solve any doubts about data collection tools and techniques. The collected information was checked for consistency and completeness on the same day by the principal investigator.

### Data management and analysis

Data were collected using an open data kit (ODK) and exported to SPSS version 25 for analysis. Descriptive statistics were summarized by frequency, percent, and mean with standard deviation. A binary logistic regression analysis was done to identify factors associated with CP. Variables with a p-value <0.25 in the bivariable analysis were included in the final multivariable analysis. An adjusted odds ratio with a 95% confidence interval was reported to show the strength of the association. The significance of the association was declared at a p-value< 0.05. The goodness of fit was checked by the Hosmer and Lemeshow test (p-value = 0.35), and it met the assumption.

### Ethical considerations

The Institutional Review Board (IRB) of Hawassa University, College of Medicine and Health Sciences granted ethical approval before data collection. An official letter was obtained from the Department of Pediatrics and Child Health to the respective bodies of the referral hospital for their kind permission and cooperation throughout the study. Due to the nature of the retrospective retrieval of patient data, the institutional review board of Hawassa University waived the requirement for an informed written consent. The personal informant was kept anonymous throughout the investigation, and information concerning particular personal identifiers, like patient names, was not collected.

## Results

### Socio-demographic characteristics

A total of 240 participants (80 cases and 160 controls) were included in the study, yielding a 96.4% retrieval rate. In the majority of controls (64.4%) and in 36 (45.4%) cases, mothers were aged between 25 and 34 years. The mean (SD) age of mothers of CP patients was 26.8 ± 12.5 years, and for controls, it was 26.7 (±13.3) years. More than half of the 84 (52.5%) cases and 38 (47.5%) controls were aged above 5 years. The mean (SD) age for cases was 5.4 ± 2.58 years, and for controls, it was 6.3 (±3.3) years. Seventy (87.5%) cases and 106 (66.2%) controls had urban residency (Table 1).

**Table 1:** Sociodemographic characteristics among pediatric CP patients at HUSCH, 2024.

Variables	Category	Case (n = 80)	Controls (n = 160)	p-value
Age of child in years	<5	42(52.5)	76(47.5)	0.465
	>5	38(47.5)	84(52.5)	
Sex	Male	48(60.0)	80(50.0)	0.143
	Female	32(40.0)	80(50.0)	
Maternal age	<25	32(40.0)	38(23.7)	0.013
	25-34	36(45.0)	103(64.4)	
	≥35	12(15.0)	19(11.9)	
Residency	Rural	10(12.5)	54(33.7)	0.002
	Urban	70(87.5)	106(66.2)	

### Prenatal history

The majority of cases(92.5%)and controls (90.6%) had attended antenatal care. Seventeen (21.2%) of cases and 18.7% of controls had a history of chronic medical illness during pregnancy. Hypertension was the most common (30.5%) chronic illness, followed by diabetes mellitus (22.03%). Over a third of cases and 6.9% of controls had a history of infection. Urinary tract infection was the most prevalent maternal infection (22.2%), followed by malaria (42.5%) and chorioamnionitis (8.5%) (Table 2).

**Table 2:** Prenatal-related characteristics among pediatric CP patients, HUSCH of Hawassa City,2024.

Variables	Category	Case (n = 80)	Controls (n = 160)	p-value
Antenatal care	Yes	74(92.5)	145(90.6)	0.628
	No	6(7.5)	15(9.4)	
Maternal chronic illness	Yes	17(21.2)	30(18.7)	0.933
	No	63(78.8)	130(81.3)	
Maternal infection	Yes	29(36.3)	11(6.9)	<0.001
	No	51(63.7)	149(93.1)	
History of CP	Yes	2(2.50)	4(2.60)	0.964
	No	78(97.50)	150(97.4)	

### Intrapartum and postpartum characteristics

Almost all (91.2%) cases and 146 (96%) controls were singletons. More than half (53.7%) of cases and 73.9% of controls were born through spontaneous vaginal delivery. Fourteen (17.5%) cases and 25 (15.6%) controls had a history of birth trauma. Sixty-seven (83.7%) cases and nine (5.6%) controls have congenital anomalies. Thirty-three (41.5%) cases and 14 (9.1%) controls had low birth weight. About two-thirds of the cases and 54 (33.7%) controls had perinatal asphyxia. Fifty-six (70%) cases and 52 (32.5%) controls had a history of prolonged labor. Thirty-five (44.3%) cases and 24 (15%) controls had meconium aspiration syndrome. About 41.2% of cases and 12 (7.5%) controls had CNS infection during infancy, and 47 (58.8%) cases and 36 (22.5%) controls had a history of neonatal infections (Table 3).

**Table 3:** Intrapartum and postpartum characteristics among pediatric CP patients at HUSCH, Hawassa City, 2024.

Variables	Category	Case	Controls	p-value
Pregnancy outcome	Singleton	73(91.2)	142(96)	0.144
	Multiple	7(8.8)	6(4.0)	
Mode of delivery	SVD	43(53.7)	123(76.9)	0.004
	Cesarean section	29(36.2)	27(13.8)	
	Instrumental vacuum	8(10.0)	10(6.3)	
Birth trauma	Yes	14(17.5)	25(15.6)	0.711
	No	66(82.5)	135(84.4)	
Congenital anomaly	Yes	67(83.7)	9(5.6)	0.007
	No	13(16.3)	151(94.4)	
Birth weight	<2.5 kg	33(41.2)	14(9.1)	<0.001
	2.5-4 kg	45(56.3)	127(82.5)	
	>4 kg	2(2.50)	13(8.4)	
Gestational age at birth	Term	51(63.7)	143(89.4)	<0.001
	Preterm	18(22.5)	10(6.2)	
	Post-term	11(13.8)	7(4.4)	
Prolonged labor	Yes	56(70)	52(32.5)	<0.001
	No	24(30)	108(67.5)	
CNS infection during infancy	Yes	33(41.2)	12(7.5)	<0.001
	No	47(58.8)	148(92.5)	
Perinatal asphyxia	Yes	54(67.5)	54(33.7)	<0.001
	No	26(32.5)	106(66.3)	
Hyperbilirubinemia	Yes	11(13.7)	5(5.0)	0.018
	No	69(86.2)	152(95)	
Meconium aspiration syndrome	Yes	35(44.3)	24(15.0)	<0.001
	No	44(55.7)	136(85.0)	
Neonatal infection	Yes	47(58.8)	36(22.5)	<0.001
	No	33(41.8)	124(77.5)	

### Pattern of cerebral palsy and treatment

Nearly two-thirds (63.7%) of children with CP had neuropsychiatric comorbidities, with epilepsy being the most common (35, 68.6%). Of all CP patients, 71.3% received treatment, and 84.2% received physiotherapy as a treatment and had follow-up in physiotherapy centers (Table 4).

**Table 4:** Pattern of cerebral palsy and treatment characteristics among pediatric CP patients at HUCSH, Hawassa City,2024.

Variables	Category	Frequency	Percentage
Neuroimaging	Yes	49	61.3
	No	31	38.7
Associated neuropsychiatric comorbidity	Yes	51	63.7
	No	29	36.3
Associated comorbidity(n = 51)	Epilepsy	35	68.6
	Microcephaly	7	13.7
	Vision/hearing impairment	9	17.6
Treatment received	Yes	57	71.3
	No	23	28.7
Physiotherapy (n = 57)	Yes	48	84.2
	No	9	15.8

**Fig 1:**
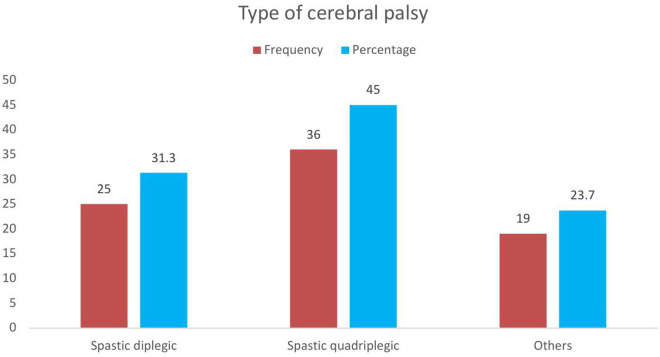
Types of CP among pediatric CP patients at HUCSH, Hawassa City,2024.

Spastic quadriplegic cerebral palsy is the most common type (36. 5%), followed by Spastic diplegic CP (31.3%) (Fig 1).

### Factors associated with Cerebral Palsy

In the bivariable logistic regression analysis, sex, maternal age, residency, maternal infection, mode of delivery, birth weight, gestational age, prolonged labor, CNS infection in infancy, perinatal asphyxia, meconium aspiration syndrome, and neonatal infection were found to have a significant association with a p-value of <0.25. In the multivariable logistic regression, maternal infection, low birth weight, prolonged duration of labor, birth asphyxia, and CNS infection during infancy were significantly associated with CP.

### Model adequacy test

**Fig 2:**
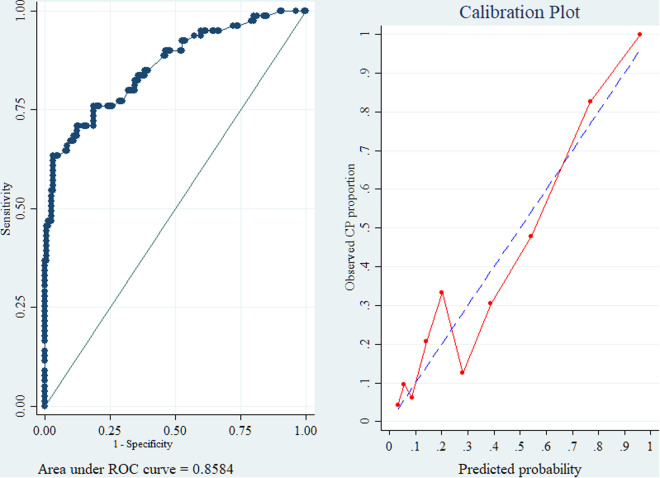
Model adequacy test for binary logistic regression on risk factors of cerebral palsy. Note: In this case–control study, the prevalence of cerebral palsy was fixed by design; therefore, predicted probabilities do not represent absolute risks. While the AUC reflects valid discrimination, calibration results should be interpreted within the study sample and not as population-level calibration.

The model demonstrated excellent discriminative ability, with the ROC curve showing an AUC of 0.8584, indicating a high capacity to distinguish between cases and controls. The calibration plot revealed that predicted probabilities closely matched observed outcomes, with the calibration curve lying near the line of perfect calibration, although slight deviations were noted at lower predicted probabilities, suggesting that the model slightly over- or under-predicts risk in those ranges. Overall, the model showed strong performance in both discrimination and calibration (Fig 2).

The odds of cerebral palsy for a child who had a mother who had an infection during pregnancy were 4 [AOR:4.1; 95% (1.39, 12.1)] times more likely compared to their counterparts. A low-birth-weight child was 4 [AOR:4.1; 95% (1.49, 11.2)] times more likely to develop cerebral palsy compared to a normal-birth-weight child. Similarly, prolonged duration of labor was 3.2 [AOR:3.2; 95% (1.47, 7.00)] times more likely to increase the odds of having a child with CP.

Children who had a history of perinatal asphyxia were nearly three [AOR: 2.65; 95% (1.06, 6.65)] times more likely to develop CP than those who had no history of perinatal asphyxia. Moreover, children who had CNS infection during infancy were 3.4 [AOR:3.4; 95% (1.21, 9.64)] times more likely to develop CP compared to those who had no history of CNS infection (Table 5).

**Table 5:** Binary logistic regression analysis of factors associated with cerebral palsy.

Variables	Category	Case	Controls	COR (95%CI)	AOR (95%CI)
Sex	Male	48	80	1.5(0.87,2.58)	1.51(0.69,3.29)
	Female	32	80	1	1
Maternal age	<25	32	38	2.40(1.31,4.40)	1.24(0.48,3.24)
	≥35	12	19	1.80(0.79,4.08)	1.12(0.33,3.76)
	25-34	36	103	1	1
Residency	Urban	70	106	3.09(1.46,6.49)	1.12(0.41,3.08)
	Rural	10	54	1	1
Maternal infection	Yes	29	11	7.7(3.58,16.5)	4.1(1.39, 12.1) ^*^
	No	51	149	1	1
Mode of delivery	SVD	43	123	1	1
	CS	29	27	3.1(1.63,5.76)	1.21(0.46,3.21)
	Instrumental	8	10	2.28(0.84,6.1)	0.25(0.04,1.32)
Birth weight	<2.5 kg	33	14	6.6(3.26,13.5)	4.1(1.49,11.2) ^**^
	>4 kg	2	13	0.43(0.09,1.99)	0.13(0.01, 1.41)
	2.5-4 kg	45	127	1	1
Gestational age	Term	51	143	1	1
	Preterm	18	10	5.0(2.18,11.6)	2.68(0.81,8.91)
	Post-term	11	7	4.4(1.62,11.9)	5.2(0.79,34.9)
Prolonged labor	Yes	56	52	4.8(2.70, 8.66)	3.20(1.47, 7.00)^**^
	No	24	108	1	1
CNS infection during infancy	Yes	33	12	8.6(4.14,18.1)	3.42(1.21,9.64)^*^
	No	47	148	1	1
Perinatal asphyxia	Yes	54	54	4(2.30,7.21)	2.65(1.06,6.65)^*^
	No	26	106	1	1
MAS	Yes	35	24	4.5(2.42,8.38)	1.51(0.57,4.02)
	No	44	136	1	1
Neonatal infection	Yes	38	45	4.9(2.74,8.75)	1.32(0.53,3.27)
	No	42	115	1	1

* Significant at a p-value <0.05 and

** significant at a p-value <0.001; AOR: Adjusted odds ratio; COR: crude odds ratio; MAS: meconium aspiration syndrome

## Discussion

The study aimed to evaluate the risk factors associated with cerebral palsy in children under 15 years old at HUCSH from July 2019 to December 2023, as understanding these factors is essential for implementing preventive measures, managing care, and providing rehabilitation services.

The study found a significant association between a child’s birth weight and cerebral palsy. Low-birth-weight children were four times more likely to develop cerebral palsy compared to normal-birth-weight children, which is comparable to studies done in China, Sweden, Brazil, Nigeria, and Arabic-speaking countries [10,20,27,28]. Low-birth-weight infants, especially those born prematurely, are particularly vulnerable to hypoxic-ischemic injury due to their immature brain structure and underdeveloped white matter [29]. They also face a higher incidence of neonatal complications such as intraventricular hemorrhage, periventricular leukomalacia, respiratory distress, sepsis, and hypoglycemia, all of which can negatively impact normal brain development [30,31]. Furthermore, nutritional deficiencies and compromised placental function, leading to intrauterine growth restriction, can impair the delivery of oxygen and nutrients to the developing brain, amplifying its vulnerability [32,33].

In this study, maternal infection and prolonged labor were shown as significant risk factors for cerebral palsy. The finding was consistent with a study done in Pakistan [11], which revealed that maternal urinary tract infection is linked with cerebral palsy. Maternal infections, especially chorioamnionitis and syphilis, might raise the risk of cerebral palsy due to probable brain damage caused by inflammation [24,34]. Prolonged labor can also raise the risk, as it may result in complications such as fetal asphyxia and neonatal sepsis and meningitis, which can harm the developing brain.

The study found that children who experience birth asphyxia are nearly three times more likely to develop cerebral palsy compared to those without a history of asphyxia. The finding was supported by a meta-analysis conducted in different Arabic-speaking countries [27]. This could be explained by the fact that birth asphyxia can result in hypoxic-ischemic encephalopathy, which may lead to permanent neurological injuries and potential neurodevelopmental disorders, such as developmental delay and cerebral palsy [35]. This underscores the importance of prevention and treatment of perinatal asphyxia.

Moreover, an infection of the central nervous system during infancy increases the risk of developing cerebral palsy. Children who experienced a CNS infection during infancy were 3.4 times more likely to develop CP compared to those without a history of CNS infection. The finding was supported by a study conducted at Ayder Comprehensive Specialized Hospital in northern Ethiopia [21] and Uganda [36]. This is because the infection can trigger inflammation and injury to the brain, resulting in chronic neurological problems such as cerebral palsy [37,38]. Children with a central nervous system infection should be closely monitored for developmental delays and motor impairment so that intervention can be initiated swiftly to improve the child’s outcomes and quality of life.

### Limitations of the study

The study was based on secondary data; hence, it was difficult to find some factors that influence the occurrence of cerebral palsy in the study population from patients’ charts. For instance, it was not possible to access data on maternal infectious or chronic diseases, the use of prenatal and perinatal drugs, consanguinity, or harmful habits such as smoking, alcoholism, and the use of other drugs. Additionally, certain clinical features (e.g., perinatal asphyxia) might have influenced the clinical suspicion of CP, though diagnosis was confirmed based on functional and neurological criteria. Moreover, the sample may be skewed because only children seen in the neurology clinic were included.

## Conclusion

This study reveals that perinatal asphyxia, maternal infection during pregnancy, low birth weight, prolonged labor, and CNS infection during infancy were significantly associated with the occurrence of CP. Public health education should focus on raising awareness about cerebral palsy, emphasizing its preventable nature and the importance of early identification and intervention. Pregnant women should receive care in well-equipped health facilities for antenatal care and delivery, and health professionals should be trained adequately to prevent and treat perinatal asphyxia. Moreover, stakeholders should strengthen interventions such as regular screening for infections in pregnant women and implementing measures to reduce the incidence of CNS infections in infants to help mitigate the occurrence of CP. Future prospective multi-center studies with broader variable inclusion would better compensate for the limitations of this study.

## Supporting information

S1 Data The dataset was analyzed for this study. (XLS)

## Abbreviations

APH: Antepartum Hemorrhage
CP: Cerebral Palsy
HIE: Hypoxic Ischemic Encephalopathy
IVH: Intra Ventricular Hemorrhage
LBW: Low Birth Weight
PNA: Perinatal Asphyxia
UTI: Urinary Tract Infection.
